# Typologies of physical education experts’ perspectives on designing physical activity program guidelines for older adults in urban–rural community centers: a Q-methodology study

**DOI:** 10.3389/fpubh.2025.1675612

**Published:** 2025-11-28

**Authors:** Goomyeung Kwon, Wonjae Jeon, Heonsu Gwon

**Affiliations:** 1 Department of Physical Education, Daegu Haany University, Gyeongsan-si, Gyeongsangbuk-do, Republic of Korea; 2 Department of Physical Education, Korea National University of Education, Cheongju-si, Chungbuk, Republic of Korea; 3 Industry-Academia Cooperation Center, Yong In University, Yongin-si, Gyeonggi-do, Republic of Korea

**Keywords:** Q methodology, physical education experts, senior centers, physical activity programs, healthy aging

## Abstract

This study employed Q methodology to identify and categorize the subjective perspectives of physical education experts on developing physical activity program guidelines for older adults in senior community centers situated in South Korea’s urban–rural hybrid regions. As health promotion for aging populations becomes increasingly important, existing physical activity programs often lack expert-led design and contextual relevance. To address this issue, 25 Q-statements were refined based on a literature review and focus group interviews. Twenty qualified experts participated in the Q-sorting process, and the data were analyzed using centroid factor extraction and Varimax rotation. The analysis revealed four distinct types: (1) infrastructure-focused planning, (2) psychosocial well-being emphasis, (3) instructor–participant relational focus, and (4) structured and accessible design. While participants’ perspectives differed, they consistently emphasized the importance of stress reduction and cognitive engagement across all types. These findings underscore the need for multidimensional, expert-informed, and locally adapted physical activity program guidelines for senior centers, particularly in transitional urban–rural areas. The results offer practical insights for developing evidence-based interventions that support healthy aging and improve the quality of life for older adults.

## 1 Introduction

As the global population ages, there has been a considerable increase in societal interest in managing aging and preventing age-related conditions, particularly in areas such as health, nutrition, and psychological well-being (1, 2). Notably, the concept of a “centenarian society” has become more widely accepted, fueled by rising life expectancy. In this context, older adults are making various efforts to maintain their health and improve their quality of life in pursuit of healthy longevity.

Health promotion in later life is recognized as a critical strategy for addressing aging-related challenges. Participation in health-promoting activities supports physical well-being and contributes to maintaining and expanding social networks. These activities foster life satisfaction and psychological well-being, facilitating the redefinition of life goals and enhancing quality of life (3, 4).

In response, many governments have developed policies that reinforce community infrastructures and services to support the health of the older adults (5, 6). South Korea has followed this trend by establishing legal and institutional frameworks, including the 2017 revision of the Senior Welfare Act, which defined 16 categories of senior welfare facilities. Senior welfare centers, among these facilities, are mandated to provide education, recreational activities, social engagement, health promotion, and disease prevention services (7). Additionally, the National Health Promotion Act has led to the nationwide expansion of community health initiatives tailored to older adults (8).

One of the key facilities within this infrastructure is the senior community center. These centers, which include welfare centers and senior clubs, serve as hubs for comprehensive services in welfare, education, leisure, and health management (9). They play a vital role in promoting physical activity and social interaction, which is central to executing community-based health promotion programs (10).

Domestic research has steadily increased in recognizing the value of senior community centers. These centers are not only leisure venues, but also core institutions that address social isolation and enhance community wellness (5). Previous studies have examined aspects such as spatial configurations, service integration models, and facility operations. For instance, comparative analyses between South Korea and Japan suggest that multifunctional integration within these centers positively influences older adults’ social participation (11). Min (12) found that physical activity services are linked to increased life satisfaction and happiness, and Kim and Kang (13) identified geographic disparities in service accessibility in rural areas.

International studies also emphasize the effectiveness of community-based senior programs. Siegenthaler and Vaughan (14) found that participation in community-based leisure and physical activities significantly contributes to preserving identity and quality of life among older adults. The World Health Organization (WHO) emphasizes structured, community-based health service delivery as a key strategy for addressing global aging trends (6). These findings support the claim that health-oriented leisure programs positively impact satisfaction and well-being (14, 15).

Nevertheless, the existing literature has largely focused on the physical accessibility of facilities and usage rates, paying limited attention to the professionalism and effectiveness of physical activity programs. Two major challenges remain. First, there is an absence of high-quality programs tailored to older adults’ physical conditions and health statuses. Many existing programs are general in nature and lack personalization or clinical relevance (16). Second, most personnel at senior community centers are social workers, administrative staff, or volunteers, with limited representation from physical education specialists (17). This deficiency substantially hinders the design and implementation of specialized, professionally structured physical activity programs (18).

This study addresses these gaps by exploring effective development strategies for physical activity programs in senior community centers located in urban–rural complex cities in South Korea. Urban–rural complex cities (도농복합시, Do-Nong-Bok-Hab-Si) are administrative regions that combine urban and rural areas within a single municipal boundary. These cities emerged due to South Korea’s rapid urbanization and administrative reforms in the 1990s, merging previously separate urban centers and surrounding rural townships into unified jurisdictions. These regions present unique challenges and opportunities for older adults health promotion. On one hand, they experience faster demographic aging than metropolitan areas because of rural depopulation and the aging-in-place phenomenon. On the other hand, they often face disparities in healthcare infrastructure, access to social services, and availability of professional staff compared to large cities. Senior community centers in these areas serve diverse populations with different health literacy levels, mobility, and cultural expectations, making standardized program design especially challenging (13). Understanding expert perspectives on program development in this transitional context is thus essential to ensure that physical activity interventions are both evidence-based and tailored to the local setting. Senior community centers are increasingly seen as a strategic solution to address aging-related physiological, psychological, and social challenges, particularly through the integration of professional physical activity services. In Korea, over 400 senior centers provide leisure programs, yet significant regional disparities in quality and access still exist (13).

This study explores the essential service elements required by current and future older adults users to inform the development of evidence-based health promotion programs.

The study explores the following research questions: First, what different subjective typologies can be identified among physical education experts regarding the development of effective physical activity program guidelines for senior community centers? Second, what are the key characteristics, underlying values, and practical implications of each identified typology?

## 2 Methods

This study used Q methodology to examine the subjective views of physical education experts regarding the development of physical activity program guidelines for senior community centers in urban–rural composite cities in South Korea. Q methodology combines the strengths of qualitative and quantitative research approaches, making it particularly well-suited for examining human subjectivity, such as individual experiences, preferences, values, and beliefs regarding a specific research topic (20, 21). It has been widely applied in various fields, such as sociology, psychology, political science, nursing, and education. Unlike traditional statistical techniques, Q methodology emphasizes individuals’ contextual perceptions and patterns, making it especially suitable for revealing subjective viewpoints (22).

### 2.1 Q-population and Q-sample

The Q-population was constructed using a dual approach to ensure theoretical rigor and empirical relevance. First, an indirect assessment of how experts in older adults physical education perceive physical activity programs was conducted through a review of existing literature and policy documents (23). Second, focus group interviews (FGIs) were conducted with key stakeholders, such as administrators of senior welfare centers and program coordinators, to obtain direct qualitative insights. These procedures resulted in the finalization of the Q-population.

Next, 42 statements, referred to as the “concourse,” were reinterpreted and systematically reclassified. Three doctoral-level experts in relevant research domains reviewed the initial pool of statements, and a panel of study participants with domain-relevant expertise validated them. During this refinement process, redundant or conceptually ambiguous statements were systematically removed to ensure conceptual clarity, distinctiveness, and alignment with the research objectives.

A final set of 25 Q-sample statements was selected. According to Kerlinger (25), when research involves complex cognitive processes, it is advisable to use a smaller Q-sample of fewer than 30 items since larger sets may compromise reliability. The 25 statements were systematically sampled from the concourse to ensure balanced coverage across infrastructure, psychosocial, relational, and programmatic dimensions. In this study, reliability was confirmed through repeated Q-sorting conducted with five participants, resulting in a correlation coefficient of *r* = 0.72 (22).

Table 1 presents the final set of Q-sample statements used in this study.

**Table 1:** Q-sample.

Q number	Q statements
1	Acquisition of new knowledge and information
2	Program content that supports daily life activities
3	Instructor expertise in the relevant subject area
4	Convenience of welfare center facilities
5	Incorporation of content requested by learners
6	Thorough preparation for program delivery
7	Opportunities for leisure and hobby engagement
8	Safety features of educational facilities
9	Clarity of program content for learners
10	Clear explanations provided by instructors
11	Fun and novel learning experiences
12	Adequate space for program activities
13	Appropriate duration of the program
14	Responsiveness to learners’ suggestions and needs
15	Health improvement through the program
16	Access to essential teaching materials and equipment
17	Linkage with community resources
18	Kindness and respect shown to learners
19	Stress reduction through participation
20	Provision of resting areas for participants
21	Diversity of program offerings
22	Instructor–learner communication about the program
23	Opportunities for forming peer groups
24	Cleanliness of educational facilities
25	Participation in community service

### 2.2 P-sample

In Q methodology, large sample sizes are unnecessary. The objective is to identify distinct subjective patterns rather than generalize to a broader population (26). Statistically, the number of participants (P-sample) should be smaller than the number of Q-statements to allow for a clear distinction of factors (27). Participants were selected based on their expertise and direct involvement in senior-focused physical activity services (28). Consistent with the small-sample principle of Q methodology (29), a total of 20 experts were recruited. These included: (1) professionals with degrees in social welfare, public health, or physical education who have at least 3 years of experience in the field, and (2) doctoral-level specialists in gerontology or senior sports science. Table 2 summarizes the demographic characteristics of the P-samples.

**Table 2:** Summary of characteristics for P sample & factor weight.

Factor (Type)	P sample	Age	Gender	Career (years)	Factor weight
Type 1. (*N* = 6)	5	57	Male	16	0.69
	6	55	Female	25	0.63
	7	58	Female	25	0.59
	8	55	Female	24	0.58
	12	59	Female	16	0.73
	19	56	Male	26	0.64
Type 2. (*N* = 4)	10	54	Female	25	0.85
	13	49	Female	25	0.87
	16	57	Female	13	0.43
	17	55	Female	20	0.65
Type 3. (*N* = 6)	2	58	Female	22	0.79
	3	51	Female	19	0.62
	9	53	Female	16	0.40
	14	53	Female	22	0.84
	18	57	Male	27	0.51
	20	58	Male	24	0.60
Type 4. (*N* = 4)	1	54	Female	23	0.54
	4	58	Male	23	0.75
	11	56	Female	28	0.69
	15	51	Female	15	0.72

### 2.3 Q-sorting and factor analysis

Q-sorting is the process by which participants rank Q-sample statements according to their personal opinions (30). In this study, participants sorted a total of 25 statements using a forced-choice distribution grid ranging from +4 (strongly agree) to −4 (strongly disagree). A nine-point quasi-normal distribution was employed, which is considered appropriate when fewer than 40 statements are used (31).

In forced sorting, the researcher predefines the number of statements that can be placed at each ranking level to ensure a balanced distribution (31). Participants initially grouped the statements into positive, neutral, and negative categories and then assigned them to the grid according to their perceived importance.

To better understand the reasoning behind their responses, open-ended responses were collected for the items ranked as +4 (most agree) and −4 (most disagree). All Q-sorting sessions were administered face-to-face.

To better understand participants’ reasoning, open-ended responses were collected for the items ranked as +4 (most agree) and −4 (most disagree). All Q-sorting sessions were administered face-to-face.

To enable quantitative analysis, scores were assigned as follows: −4 = 1 point, −3 = 2 points, −2 = 3 points, −1 = 4 points, 0 = 5 points, +1 = 6 points, +2 = 7 points, +3 = 8 points, and +4 = 9 points.

**Figure 1:**
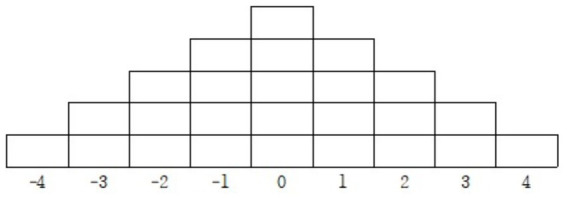
Q sorting response table.

The overall sorting framework is illustrated in Figure 1.

### 2.4 Data processing

The Q-sorting data were analyzed using PQMethod version 2.35 (32). Factor extraction was performed using the centroid method, followed by Varimax rotation for clarity. Based on the Kaiser–Guttman criterion, only factors with eigenvalues ≥ 1.0 were retained (33, 34). Factors were rotated using orthogonal Varimax rotation to maximize interpretability and ensure that each factor represented a distinct subjective viewpoint. The optimal number of factors was determined by testing solutions ranging from two to seven iteratively (33, 34). A participant was defined as significantly loading on a factor if the absolute loading value exceeded 0.40, consistent with standard Q-methodology conventions (31).

## 3 Results

### 3.1 Eigenvalues (EVs), variance, and correlation among guideline types for developing physical activity programs for older adults

Based on the results of the factor analysis, four distinct types were identified. The respective eigenvalues were 5.6719, 2.3674, 1.8981, and 1.7235. Each type explained 28, 12, 9, and 9% of the variance, respectively, for a cumulative total of 59%. These values meet the Kaiser–Guttman criterion, which retains only factors with eigenvalues ≥ 1.0 (33, 34).

Table 3 presents the detailed eigenvalues and variance explained for each type.

**Table 3:** Eigenvalue (EVs) and variance between types.

	Type 1	Type 2	Type 3	Type 4
Eigenvalue (EVs)	5.6719	2.3674	1.8981	1.7235
% of explanatory variance	0.28	0.12	0.09	0.09
Total variance	0.28	0.40	0.49	0.58

Table 4 displays the correlation coefficients among the four types. All correlations were positive, suggesting an overall tendency toward shared perspectives. However, the relatively low coefficients also indicate that the types are conceptually distinct. Notably, Types 2 and 4 showed the weakest correlation (r = 0.1542), implying the greatest differentiation between them. This divergence may reflect fundamentally different priorities: Type 2 emphasizes psychosocial and emotional dimensions (e.g., stress relief, leisure engagement), which are intrinsic and participant-centered, whereas Type 4 prioritizes structural clarity and systematization (e.g., program duration, content clarity), which are extrinsic and design-centered. These opposing orientations—one focusing on affective experience and the other on procedural organization—may explain the minimal overlap between the two perspectives.

**Table 4:** Correlations between types.

	Type 1	Type 2	Type 3	Type 4
Type 1	1.000			
Type 2	0.2358	1.000		
Type 3	0.4001	0.2273	1.000	
Type 4	0.4120	0.1542	0.2272	1.000

### 3.2 Characteristics of each identified type

#### 3.2.1 Type 1 (*n* = 6): “infrastructure-focused program planning”

Participants in Type 1 placed a high priority on safety features of educational facilities (Q8), program content that supports daily life activities (Q2), and the convenience of welfare center facilities (Q4). These preferences suggest a strong emphasis on physical infrastructure and practical relevance in program design.

The lowest-ranked statements included participation in community service (Q25), opportunities for forming peer groups (Q23), and linkage with community resources (Q17). This indicates relatively low concern for external collaboration or community engagement.

This type accounted for the largest group of participants and had the highest factor loadings from P12 (0.73), P5 (0.69), P19 (0.64), and P6 (0.63).

The detailed Z-score distribution and representative Q statements for this type are presented in Table 5.

**Table 5:** Statements with a *Z* score of ±1.00 or higher for each type and *Z* score results.

	Q statement		Z score
Type 1	Positive	Q 8	1.857
		Q 2	1.638
		Q 4	1.413
	Negative	Q 17	−1.307
		Q 23	−1.363
		Q 25	−2.037
Type 2	Positive	Q 19	2.020
		Q 7	1.582
		Q 2	1.393
	Negative	Q 23	−1.427
		Q 17	−1.543
		Q 10	−1.785
Type 3	Positive	Q 22	2.161
		Q 14	1.527
		Q 3	1.467
	Negative	Q 7	−1.389
		Q 15	−1.411
		Q 25	−1.962
Type 4	Positive	Q 13	2.311
		Q 9	1.140
	Negative	Q 11	−1.378
		Q 15	−1.390
		Q 25	−2.100

#### 3.2.2 Type 2 (*n* = 4): “psychosocial well-being emphasis”

Type 2 participants showed the strongest agreement with Q19 (Stress reduction through participation), Q7 (Opportunities for leisure and hobby engagement), and Q2 (Program content that supports daily life activities). These reflect a strong emphasis on emotional wellness and lifestyle enhancement.

The least agreement was recorded for Q10 (Clear explanations provided by instructors), Q17 (Linkage with community resources), and Q23 (Opportunities for forming peer groups), indicating lesser concern for external structuring and instructional clarity.

This type included four participants, with the highest factor weights observed for P13 (0.87) and P10 (0.85).

A comprehensive breakdown of the Z-score distribution and associated Q-statements is shown in Table 5.

#### 3.2.3 Type 3 (*n* = 6): “instructor-participant relational focus”

Participants classified as Type 3 placed the greatest importance on instructor-learner communication about the program (Q22), reflecting a strong preference for interpersonal engagement and instructional interaction.

As with the other types, participation in community service (Q25) was rated the lowest, followed by lower scores for opportunities to form peer groups (Q23).

This type consisted of six participants, with the most significant loadings observed for P14 (0.84), P2 (0.79), and P3 (0.62).

The detailed Z-score distribution and representative Q statements for this type are presented in Table 5.

#### 3.2.4 Type 4 (*n* = 4): “structured and accessible program design”

Participants of Type 4 emphasized structural clarity. The highest scores were assigned to Q13 (Appropriate duration of the program) and Q9 (Clarity of program content for learners), which highlight the importance of clearly defined, easy-to-follow formats.

At the lower end, the least favored item was Q25 (Participation in community service), consistent with the patterns observed in other types.

This type also comprised four participants, with the strongest factor loadings for P4 (0.75) and P15 (0.72).

Table 5 presents the key statements and standardized scores that define this type.

#### 3.2.5 Consensus across all types

Table 6 summarizes the statements that received positive consensus across all identified types. Notably, Q19 (Stress reduction through participation) and Q1 (Acquisition of new knowledge and information) were consistently regarded as highly important across all groups.

**Table 6:** Consensus statements.

Q statement	Z score
Q 19	0.67
Q 1	0.33
Q 24	−0.49
Q 16	−0.88

Conversely, Q24 (Cleanliness of educational facilities) and Q16 (Access to essential teaching materials and equipment) received consistently lower ratings, suggesting a consensus that physical conditions are less critical to program effectiveness.

## 4 Discussion

This section discusses the four distinct types of physical activity program guidelines for older adults identified in this study, drawing implications from each.

Type 1 was identified as the most prevalent, accounting for the largest proportion of participants. This prevalence suggests a widespread consensus among experts on the importance of considering facilities when planning programs. This finding aligns with the Ecological Model of Active Aging (35), which emphasizes that the built environment plays a critical role in facilitating physical activity participation among older adults. Accessible and well-maintained infrastructure reduces environmental barriers and supports sustained engagement. It indicates that such considerations are essential for developing physical activity programs that promote the health of the older population. In other words, program developers and related experts prioritize the availability, accessibility, and quality of physical infrastructure when planning and implementing these programs.

Therefore, it can be inferred that providing modern, age-appropriate, well-equipped exercise facilities is crucial for encouraging older adults living in urban–rural complex cities to participate in physical activity. In South Korea, these hybrid regions are becoming popular residential areas for older population.

As many rural areas face accelerated demographic decline and associated challenges, older individuals are relocating to urban environments in search of better health services and social integration opportunities. In this context, expanding and modernizing physical activity infrastructure in urban–rural complex cities should be considered a fundamental strategic approach.

Furthermore, ensuring the safety of older adults must be a core principle in designing and constructing any exercise facility. Every effort should be made to create accessible, inclusive spaces tailored to the physical and cognitive characteristics of the aging population (36).

Participants categorized as Type 2 emphasized that physical activity programs for older adults should prioritize psychological stability and social well-being. Statements such as Q19 (stress reduction through participation) and Q7 (opportunities for leisure and hobby engagement) received the highest ratings, reflecting the belief that mental and emotional relief is just as essential as physical fitness in program design. This perspective is strongly supported by Self-Determination Theory (SDT) (37), which posits that intrinsic motivation, autonomy, and psychological well-being are essential drivers of sustained physical activity. Programs that address emotional relief and social connectedness fulfill the psychological needs of competence, relatedness, and autonomy, thereby enhancing adherence and satisfaction among older adults (38).

This perspective is supported by a growing body of international literature. For example, Xue and Wen (39) discovered that dance-based physical activity significantly enhanced emotional well-being and self-reported health among middle-aged and older women in China. Their findings suggest that emotionally engaging and socially interactive programs foster greater satisfaction and health-related quality of life.

Similarly, Bashardoust et al. (40) emphasized the role of social support and lifestyle-based interventions in mitigating psychological distress and perceived social isolation. Their structural model of older adults well-being showed that participation in physically and socially stimulating activities can buffer feelings of loneliness and emotional decline.

Furthermore, Lin et al. (41) demonstrated that psychosocially oriented physical activity programs increased the motivation of older adult men to participate in community life. Programs integrating emotional engagement and social connection were shown to enhance participation and sustainability in later life.

Converging evidence affirms that psychosocial well-being is a core outcome, not a secondary benefit, of physical activity programming for older populations. Therefore, when developing physical activity guidelines, a holistic approach should be adopted to ensure that programs address physical competence, emotional well-being, and social enrichment, especially in communities facing aging-related vulnerabilities.

Participants in Type 3 placed a distinct emphasis on the qualities and relational competencies of instructors rather than on the program’s structural or physical components. This finding is consistent with Social Support Theory (42), which highlights the importance of interpersonal relationships in promoting health behaviors. Instructors who provide emotional, informational, and appraisal support can significantly enhance participants’ self-efficacy and program adherence (43). Moreover, Relational Pedagogy emphasizes that learning and engagement are co-constructed through respectful, empathetic, and responsive instructor-participant interactions (44). This suggests that the success of physical activity initiatives targeting older adults is closely linked to interpersonal dynamics, particularly instructors’ ability to respond effectively and build rapport.

This interpretation aligns with recent scholarship that reframes instructor–participant interaction as a core mechanism of engagement and adherence, not merely as a delivery channel. Effective programs are significantly shaped by the emotional labor and communicative attentiveness of facilitators, rather than focusing solely on physical routines.

For example, studies have shown that older adults’ program retention rates are significantly improved by the presence of instructors who foster reciprocal trust and relational continuity (45). In such contexts, communication is about more than just transferring instructions; it’s about co-creating a safe and empowering space that respects the psychological vulnerabilities and social needs of the aging population.

Furthermore, pedagogical models in aging-focused physical education increasingly emphasize affective pedagogy—instruction that is emotionally attuned, motivational, and personalized. This implies that program outcomes are often enhanced not because of what is taught, but how it is experienced relationally. Therefore, when designing physical activity programs, attention must be paid not only to the content and delivery structure but also to the personnel responsible for relational leadership within aging populations.

Participants classified as Type 4 demonstrated a clear preference for professionally structured exercise programs that older adults can easily execute. This perspective reflects the principles of Social Cognitive Theory (SCT) (46), particularly the concept of perceived behavioral control. Clear, structured, and progressively designed programs enhance participants’ confidence in their ability to complete tasks, thereby increasing self-efficacy and long-term adherence. High agreement with statements such as Q13 (appropriate program duration) and Q9 (clear program content) indicates an emphasis on programs that are theoretically sound, feasible, systematic, and tailored to aging populations.

This suggests that older adults benefit most from well-designed, evidence-based interventions rather than general recreational activities. Structured exercise protocols, especially those carefully tailored in terms of duration, intensity, and clarity, have been shown to improve physical fitness and adherence to programs.

Recent studies support this perspective. For instance, Gene-Morales et al. (47) conducted a randomized controlled trial that revealed older adults who participated in a 10-week structured fitness program experienced significant improvements in muscular strength, endurance, and balance. The study underscores the importance of clarity and standardization in achieving physical improvements. Similarly, Godhe (48) found that, even in clinical settings, professionally structured physical activity plans led to better outcomes in joint flexibility, cardiovascular health, and lean body mass than spontaneous or unstructured movement did. These benefits were especially notable in participants with low initial fitness levels.

Type 4’s focus on structured design reflects a strategic approach toward measurable, protocol-driven exercise formats that emphasize safety, clarity, and progressive adaptation—crucial factors for the aging population in urban–rural complex cities. Future programs should incorporate step-by-step instructions, standardized routines, and elements of physical literacy to boost engagement and results.

Lastly, the consensus analysis across all types revealed two shared perceptions. First, all participants agreed with Q19 (stress reduction through participation) and Q1 (acquisition of new knowledge and information). These results suggest that professionals value physical activity programs for their ability to support physical health, provide cognitive stimulation, and offer emotional relief for older adults.

These findings align with earlier research showing that structured physical activity reduces stress and supports cognitive and emotional well-being in later life (49, 50). Second, all groups consistently rated Q24 (cleanliness of educational facilities) and Q16 (availability of necessary teaching materials and equipment) as less important. This suggests that, although environmental conditions are somewhat relevant, they are not considered central elements in designing effective programs for older adults. This interpretation is consistent with Franco et al. (51), who found that program content and interpersonal interaction influence participation more than physical space or available resources.

## 5 Conclusion

The objective of this study was to identify the subjective perspectives of physical education experts regarding the development of physical activity program guidelines for older adults in senior community centers in urban–rural complex cities. Using Q methodology, four types of expert perspectives were identified, each emphasizing different priorities ranging from facility optimization to psychosocial support, communicative instruction, and structured program design.

The findings underscore the need for differentiated, evidence-based approaches to program development for older adults. While the identified perspectives varied, all groups agreed on two priorities: reducing stress and acquiring new knowledge. These insights suggest that effective physical activity programs for older adults should promote not only physical health but also emotional well-being and cognitive stimulation.

Furthermore, the study revealed that physical infrastructure and expert-led communication, particularly instructor engagement and clarity, are key to sustained participation. Program designers should consider not only the activities included but also the instructors and the structure of relational interactions.

Despite these contributions, the study has certain limitations. First, the relatively small sample size—an inherent characteristic of Q methodology—limits the generalizability of the findings. Additionally, all participants were recruited from a specific region in South Korea, which may limit geographical and professional diversity. Second, since the perspectives were collected exclusively from experts, the voices of the older adult participants were not included. Third, focusing on South Korea’s urban–rural complex regions may limit the generalizability of the findings to other cultural or demographic contexts.

Based on these findings, several directions for future research are proposed. First, follow-up studies could incorporate program participant perspectives to triangulate expert insights with actual user experiences. Future research should also include experts from broader geographical regions and diverse professional backgrounds, including social workers, public health practitioners, and gerontologists, to enhance representativeness. Second, additional qualitative research could explore the subtle differences underlying each identified perspective. Third, applying techniques such as the Delphi method could translate these expert views into concrete program modules that can be implemented in practice.

## Data Availability

The original contributions presented in the study are included in the article/supplementary material, further inquiries can be directed to the corresponding authors.
